# Site-directed mutagenesis of soybean *PEAPOD* genes using the CRISPR/Cas9 system alters tissue developmental transition

**DOI:** 10.5511/plantbiotechnology.23.0628a

**Published:** 2023-09-25

**Authors:** Jaechol Sim, Yuhei Kanazashi, Tetsuya Yamada

**Affiliations:** 1 Graduate School of Agriculture, Hokkaido University, Kita 9 Nishi 9, Kita-ku, Sapporo, Hokkaido 060-8589, Japan

**Keywords:** CRISPR/Cas9, *Glycine max*, organ enlargement, PEAPOD, SPIRAL1-LIKE 5

## Abstract

In general, plant organ size is determined using cell number and expansion. In our previous study, we generated soybean (*Glycine max*) mutants of the PEAPOD (*PPD*) genes *GmPPD1* and *GmPPD2* using the clustered regularly interspaced short palindromic repeat (CRISPR)/CRISPR-associated endonuclease 9 system. Some of these mutants exhibited extremely abnormal phenotypes, such as twisted pods and limited seeds. These phenotypes were attributed to the frameshift mutation in both *GmPPD* loci. In this study, the physiological and molecular biological properties of mutant plants with two knocked-out *GmPPD* loci (*ppd*-*KO*) were characterized. The *ppd-KO* mutant exhibited a delayed growth phase from the time of development of the unifoliolate leaves to that of first trifoliolate leaves and a stay-green phenotype, which were not observed in the other mutants of soybean or *ppd* mutants of other plant species. Gene expression analysis revealed considerably decreased expression of SPIRAL1-LIKE 5 (*GmSP1L5*), mainly causing the twisted pod phenotype observed in the *ppd-KO* mutant. The relationship between PPD and SP1L5 has not been previously reported, and in this study, we showed that that loss of PPD functioning affects *SP1L5* expression in soybean. In this study, we revealed that the decrease in PPD function contributed to organ enlargement and that complete knockout of *PPD* has a negative effect on soybean organogenesis.

## Introduction

Enlargement of plant organs can lead to an increase in plant biomass. In general, plant organ size is determined using cell number and expansion. Spontaneous or artificial mutations can result in increasing size of plant organs. Mutations in PEAPOD (PPD), a transcription factor for genes involved in cell proliferation, are closely associated with organ enlargement. The locus of *PPD* was identified for the first time in a mutant line of Arabidopsis (*Arabidopsis thaliana*); this mutant had increased leaf lamina size and dome-shaped leaves (White 2006). Because *PPD* overexpression promotes the initial arrest of mitotic cell proliferation during leaf and silique development and reduces leaf lamina size, the increased lamina growth observed in the *ppd* mutant was attributed to the increased duration of mitotic cell proliferation during leaf development (White 2006).

Loss-of-function mutations in *PPD* have been also observed in mutants of other plant species. The *Medicago truncatula* big seeds1-1 (*mtbs1-1*) mutant exhibits larger seeds and leaf tissues than its wild-type counterpart (Ge et al. 2016). In a *Vigna mungo* multiple organ gigantism (*mog*) mutant, although the total seed weight was not considerably different from that of the wild-type plant, the weight of 100 seeds was approximately 1.7 times higher than that of the wild-type plant (Naito et al. 2017). Map-based cloning of both mutants revealed that the genetic causes were mutations at the *PPD* locus (Ge et al. 2016; Naito et al. 2017). The orthologs of *PPD* (*GmPPD1* and *GmPPD2*) were knocked down in transgenic soybean (*Glycine max*) plants using RNA interference (RNAi) or artificial microRNA (amiRNA) to elucidate the molecular mechanism of the *PPD* mutations found in the *mtbs1-1* and *mog* mutants (Ge et al. 2016; Naito et al. 2017). Knock down of *GmPPD1* and *GmPPD2* resulted in enlarged seed and leaf tissue phenotype (Ge et al. 2016; Naito et al. 2017). Gene expression analysis of *PPD* knockdown-plants, using amiRNA technology, revealed that GROWTH-REGULATING FACTOR 5 (*GRF5*), GRF-INTERACTING FACTOR 1 (*GIF1*), and CYCLIN D3 (*CYCD3;3*) were noticeably upregulated in these plants compared with their expression in control plants (Ge et al. 2016). These genes are known to be closely associated with organ development in Arabidopsis (Baekelandt et al. 2018; Gonzalez et al. 2010, 2015; Kim et al. 2003). These functions of GRF, GIF, and CYCD3;3 are in line with the finding of Ge et al. (2016) that *PPD* knockdown in transgenic soybean plants elevates the expression of *GRF5*, *GIF1*, and *CYCD3;3* and enlarges seed and leaf tissues.

In our previous study, we performed site-directed mutagenesis of two *PPD* genes using the clustered regularly interspaced short palindromic repeat (CRISPR)/CRISPR-associated endonuclease 9 (Cas9) system in soybean (Kanazashi et al. 2018). We revealed that site-directed mutagenesis of *GmPPD1* and *GmPPD2* resulted in various changes in the morphological characteristics of soybeans (Kanazashi et al. 2018). We divided the double mutant plants into two main types based on their morphological characteristics. One type had rippled trifoliolate leaves that were thicker and deep green in color, had longer petioles, bigger pods, and bigger seeds than wild-type plants (Kanazashi et al. 2018). The other type had dome-shaped trifoliate leaves and extremely twisted pods with remarkably low seed yields (Kanazashi et al. 2018). The second phenotype was due to both *GmPPD* loci being knocked out with frameshift mutations (Kanazashi et al. 2018), but the molecular mechanisms underlying the phenotype without tissue enlargement remain unknown. Understanding the molecular biology and physiology of these phenotypic differences will provide novel insights into PPD function in soybean.

In this study, we selected plants from the mutant soybean line generated by Kanazashi et al. (2018) with both *GmPPD* loci knocked-out and analyzed their physiological characteristics. Transcriptome analysis of these plants was also performed. Our study provides novel insights into the physiological and molecular biological characteristics of the *ppd* mutant plants in soybean.

## Materials and methods

### Plant materials

Soybean *ppd* mutant lines were generated using the CRISPR/Cas9 system as previously described (Kanazashi et al. 2018). The gene IDs Glyma.10G244400 and Glyma.20G150000 corresponded to the *GmPPD1* and *GmPPD2* loci, respectively. We selected mutant plants that had dome-shaped trifoliate leaves and prominently twisted pods with markedly low seed yields as the plant material. Because these mutants produced very few seeds, it was difficult to maintain these mutants through subsequent generations. We found that the later generation mutant plants of the mutant line generated by Kanazashi et al. (2018) could be segregated based on two different phenotypes (bigger pods, and bigger seeds than wild-type plants, or dome-shaped trifoliate leaves and extremely twisted pods with extremely low seed yields). Therefore, the progenies of this mutant were used as the plant materials. When mutant alleles in both *GmPPD* loci were homozygous of frameshift mutations, the mutant plants exhibited the phenotype of prominently twisted pods with considerably reduced seed yield. Therefore, selection for homozygosity of frameshift mutant alleles at both *GmPPD* loci was performed for every generation. The Japanese soybean variety Kariyutaka was used as the control plant. The mutant lines and control plants were grown in a closed greenhouse at 26°C.

### Genotyping of the mutants at the *GmPPD* loci

The genotypes of the mutants at the *GmPPD* loci were confirmed via sequencing of each generation of the mutant plants. Genomic DNA was extracted from leaf pieces (approximately 5 mm×5 mm) or mature seeds according to the method described by Sugano et al. (2020). PCR fragments were amplified using specific primers (Supplementary Table S1) and sequenced either directly or after cloning into the pGEM-T-Easy vector (Promega, Madison, WI, USA) using the Big Dye Terminator Cycle method with the ABI3100 or ABI3130 Genetic Analyzer (Thermo Fisher Scientific, Waltham, MA, USA). DNA sequencing was performed by the Instrumental Analysis Division of the Graduate School of Agriculture, Hokkaido University.

### Evaluation of the morphological and physiological characteristics of the mutants

The growth period was divided into three stages. The first growth phase was from sprouting to the development of unifoliolate leaves. The secondary growth phase was from the development of the unifoliolate leaves to that of the first trifoliolate leaves. The third growth phase was from the development of the first trifoliolate leaves to the reproductive R3 stage (1 cm-sized pod). The size of the unifoliolate leaves of the mutant lines was measured, and leaf area was determined as leaf size multiplied by the length and width of the unifoliolate leaf. SPAD values of the first trifoliolate leaves were measured using a chlorophyll analyzer every 2 days from 14 to 36 days after flowering (DAF). The cell size of the unifoliolate leaves was determined. Briefly, manicure was applied to the surface of unifoliolate leaves and allowed to dry. Then, the epidermal cells in the leaves were peeled off along with the manicure and observed under the BX50 optical microscope (Olympus, Tokyo, Japan). The cell size of the epidermal cells in the microscopic images was measured using ImageJ software (https://imagej.nih.gov/ij/index.html).

### RNA-seq analysis

For transcriptome analysis, total RNA was extracted from the 1 cm-sized pods of *ppd-KO* and control plants using the LiCl precipitation method (Adachi et al. 2021). The analysis was performed without any biological replicates. Sequencing samples were prepared and paired-end sequencing using NovaSeq 6000 (Illumina, CA, USA) was performed by Rhelixa Inc. (Tokyo, Japan). The sequences were mapped with a *Glycine max* reference (*Glycine max* Wm82.a2.v1) from Phytozome (https://phytozome-next.jgi.doe.gov/info/Gmax_Wm82_a2_v1 (Accessed Feb 20, 2020)) using Bowtie 2.3.5.1 (Langmead and Salzberg 2012). Splice junctions were mapped using TopHat 2.1.1 (Kim et al. 2013). Differentially expressed genes were identified using Cufflinks 2.2.1 (Trapnell et al. 2012). A false discovery rate of Q<0.05 was considered as the threshold for differently expressed genes. The descriptions of differently expressed genes were explored via PhytoMine (https://phytozome-next.jgi.doe.gov/phytomine/bag.do?subtab=upload (Accessed Feb 20, 2020)).

### Gene expression analysis via qRT-PCR

qRT-PCR was performed in a 20 µl reaction volume containing 9.2 µl of diluted cDNA solution, 0.8 µl of each primer (1 µM), and 10 µl of TB Green® Premix Ex Taq™ II (Tli RNaseH Plus) (Takara Bio, Shiga, Japan). PCR was performed using the CFX96 Real-Time System (Bio-Rad, California, USA). The amplification conditions were as follows: 40 cycles at 95°C for 30 s, 56°C for 30 s, and 72°C for 30 s. The specificity of the amplification was verified via melting curve analysis. The expression of each gene was normalized to that of *Bic-C2* (Glyma.03G064800) using a specific primer set for each gene (Supplementary Table S1).

### Statistical analysis

Tests of significance among means of data were performed using the Student’s *t*-test. A *p*-value of <0.05 was considered statistically significant.

## Results

### Molecular characterization of mutants

Sequences of both *GmPPD* loci in the mutant plants were determined. Two mutant alleles were detected at each *GmPPD* locus in the mutant plants that segregated the phenotypes in the later generations (Table 1). At the *GmPPD1* locus, 7- and 39-nucleotide deletions were detected (Table 1). In contrast, 2- and 45-nucleotide deletions were confirmed at the *GmPPD2* locus (Table 1). The mutant plants that exhibited dome-shaped trifoliate leaves and appreciably twisted pods with markedly low seed yields harbored homozygous mutant alleles with 7- and 2-nucleotide deletions at the *GmPPD1* and *GmPPD2*, respectively. All of these mutant alleles carried frameshift mutations (Supplementary Figure S1). This mutant line was designated as *ppd-KO*. On the contrary, the mutant that harbored a homozygous frameshift mutant allele (7-nucleotide deletion) at the *GmPPD1* locus and a homozygous in-frame mutant allele (45-nucleotide deletion) at the *GmPPD2* locus was designated as *ppd1* (Table 1, Supplementary Figure S1). The *ppd2* mutant plant harbored a homozygous in-frame mutant allele (39-nucleotide deletion) at the *GmPPD1* locus and a homozygous frameshift mutant allele (2-nucleotide insertion) at the *GmPPD2* locus (Table 1, Supplementary Figure S1). In our study, site-directed mutagenesis of the *GmPPD* genes did not yield mutants in which either *GmPPD* locus was wild-type, and all plants harbored mutant alleles at the both *GmPPD* loci (Kanazashi et al. 2018). The *ppd1* and *ppd2* mutants possessing partial PPD functions were used as plant materials in this study; the in-frame mutation might have altered the original PPD function.

**Table 1:** Table 1. Alleles of the mutants at the *GmPPD* loci.

Mutant lines	*GmPPD1* locus	*GmPPD2* locus	Morphological evaluation^1^
*ppd1*	7-nucleotide deletion	45-nucleotide deletion	Moderate phenotype alteration
*ppd2*	39-nucleotide deletion	2-nucleotide insertion	Moderate phenotype alteration
*ppd-KO*	7-nucleotide deletion	2-nucleotide insertion	Severe phenotype alteration

^1^Morphological evaluation was based on the characteristics described by Kanazashi et al. (2018).

### Morphological characteristics of the mutants

**Figure 1:**
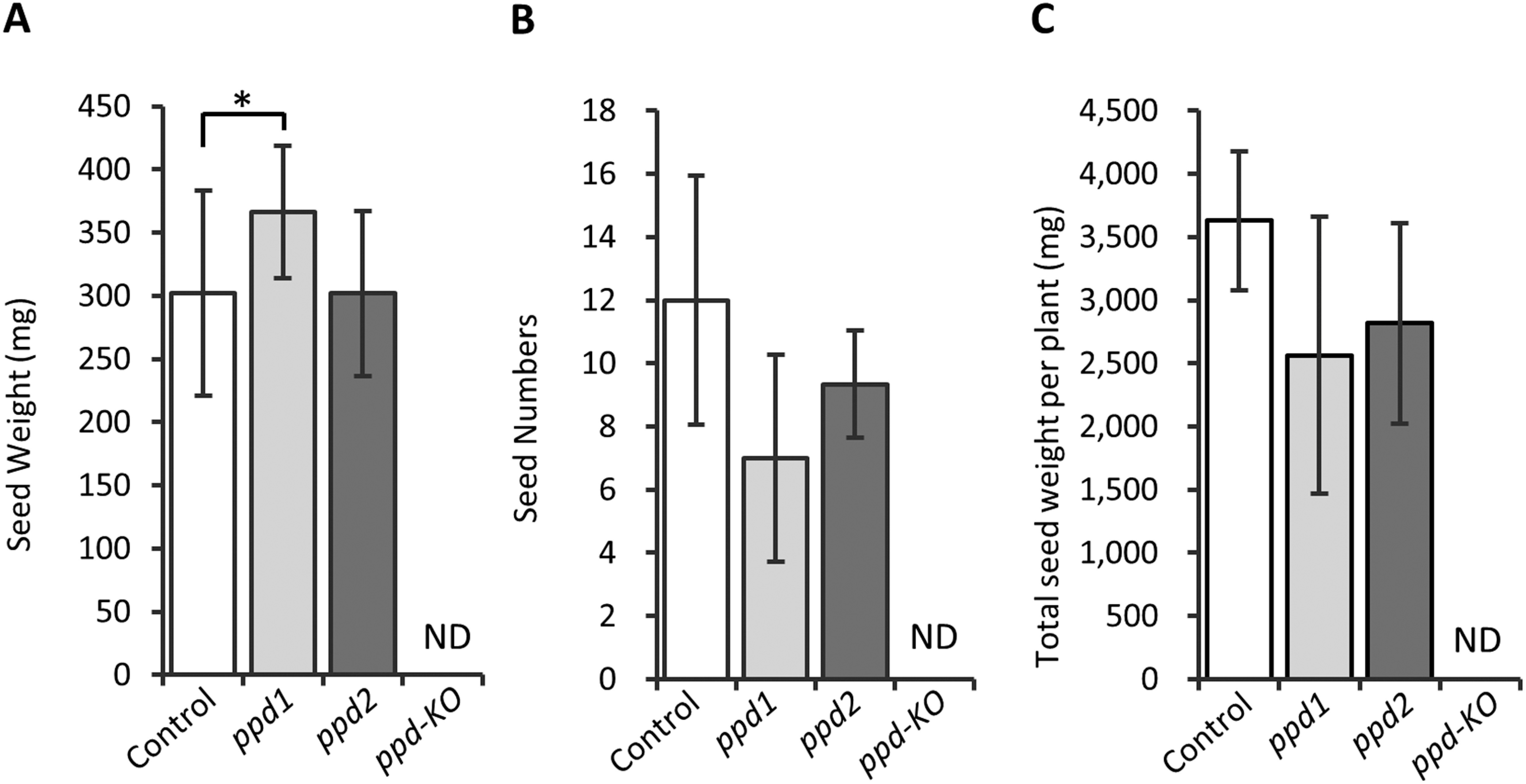
Figure 1. Seed traits of the mutants and control plants. (A) Seed weight of control plants and the *ppd1*, *ppd*2, and *ppd-KO* mutants. (B) Total seed number of control plants and the *ppd1*, *ppd*2, and *ppd-KO* mutants. (C) Total seed weight per plant of control plants and the *ppd1*, *ppd*2, and *ppd-KO* mutants. * indicates significant differences between control plants and mutants at the 5% level. All data shown are the mean±SE of 5–9 plants. ND denotes not determined.

The size of the mature seeds of the *ppd2* mutant was similar to that of control plants (Figure 1A). The seed weight of the *ppd1* mutant was significantly (*p*<0.05) higher than that of the *ppd2* mutant and control plants (Figure 1A). Further, the seed weight of the *ppd1* mutant was approximately 21% higher than that of control plants. In contrast, the number of seeds in the *ppd1* mutant was reduced to approximately 42% of that in control plants (Figure 1B). However, no differences in total seed weight were observed between the *ppd1* and *ppd2* mutants and control plants (Figure 1C). The *ppd-KO* mutant plants rarely produced seeds (Figure 1A).

**Figure 2:**
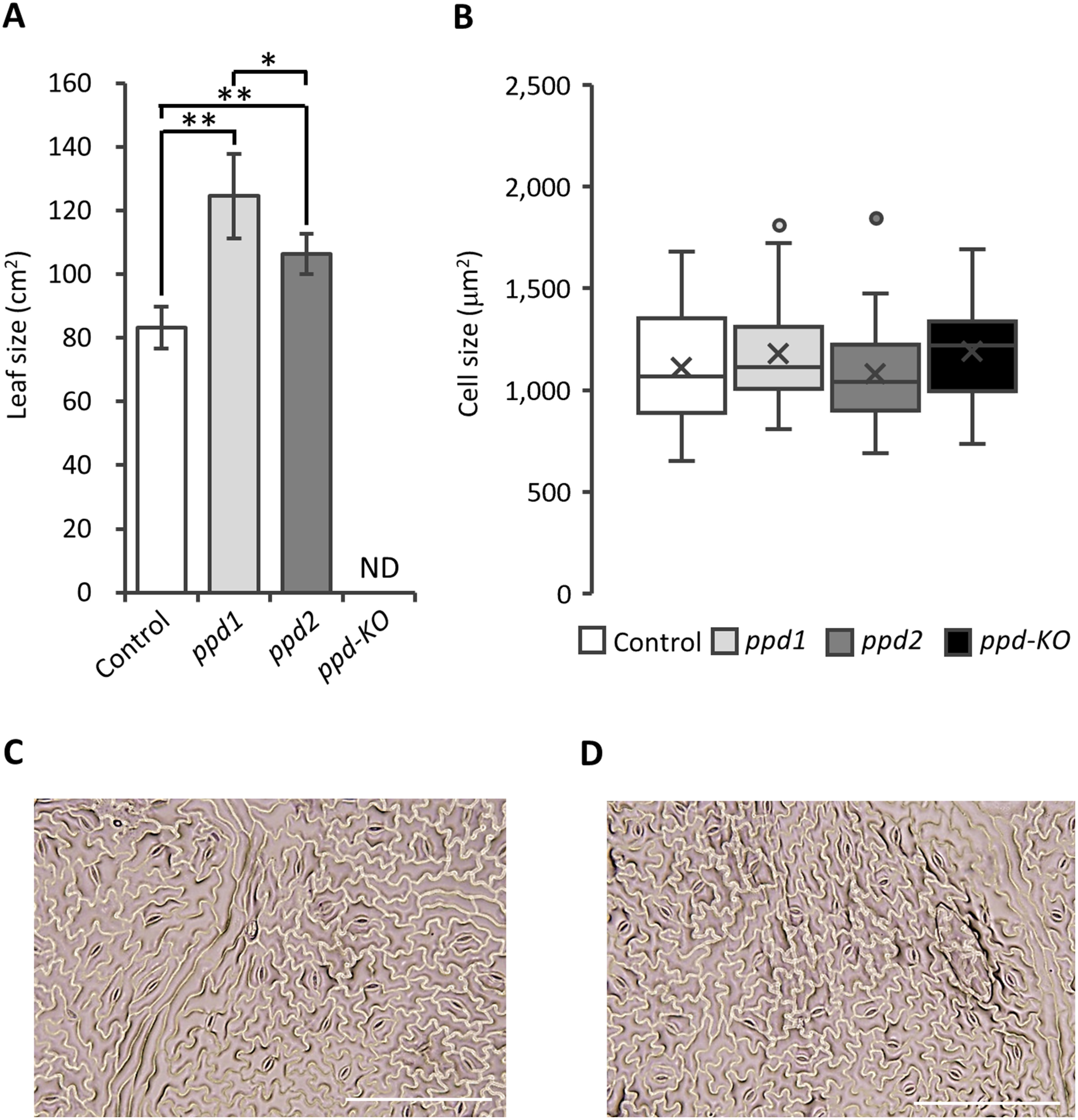
Figure 2. Sizes and epidermal cells of the unifoliolate leaves of control plants and the *ppd* mutants. (A) Sizes of the unifoliolate leaves of control and mutant plants. The unifoliolate leaves of the *ppd-KO* mutant were wrinkled and domed-shaped; therefore, the vertical and horizontal widths were not measured. * and ** indicate significant differences between control plants and the mutants at the 5% and 1% levels, respectively. All data shown are the mean±SE of 6–7 plants. ND denotes not determined. (B) Size of the epidermal cells in control plants and the mutants. The size of the epidermal cells is expressed in a boxplot diagram. Every 10 cells were measured at five areas per one unifoliolate leaf. All data are collected from 6–7 plants. (C) Epidermal cells in control plants. (D) Epidermal cells in the *ppd-KO* mutant. Scale bars: 200 µm.

The vertical and horizontal widths of the unifoliolate leaves of the mutants and control plants were measured, and the multiplied values were considered as leaf area. The mutants exhibited different unifoliolate leaf sizes. The *ppd1* mutant had the largest unifoliolate leave sizes (Figure 2A). The leaf size of the *ppd1* mutant increased by approximately 50% compared with that of control plants. Further, the size of the unifoliolate leaves of the *ppd2* mutant was larger than that of control plants (Figure 2A). The size increased by approximately 28% compared with that of control plants (Figure 2A). The unifoliolate leaves of the *ppd-KO* mutant were wrinkled and domed-shaped; therefore, vertical and horizontal widths could not be accurately measured (Figure 2A). Epidermal cells were isolated from unifoliolate leaves, and cell size was measured using ImageJ. The size of the epidermal cells in control plants was approximately 1,110 µm^2^. However, no differences were observed in the size of the epidermal cells in the mutants (Figure 2B). Furthermore, the shape of the epidermal cells was similar in control plants and the *ppd-KO* mutant (Figure 2C, D).

### Physiological characteristics of the mutants

**Figure 3:**
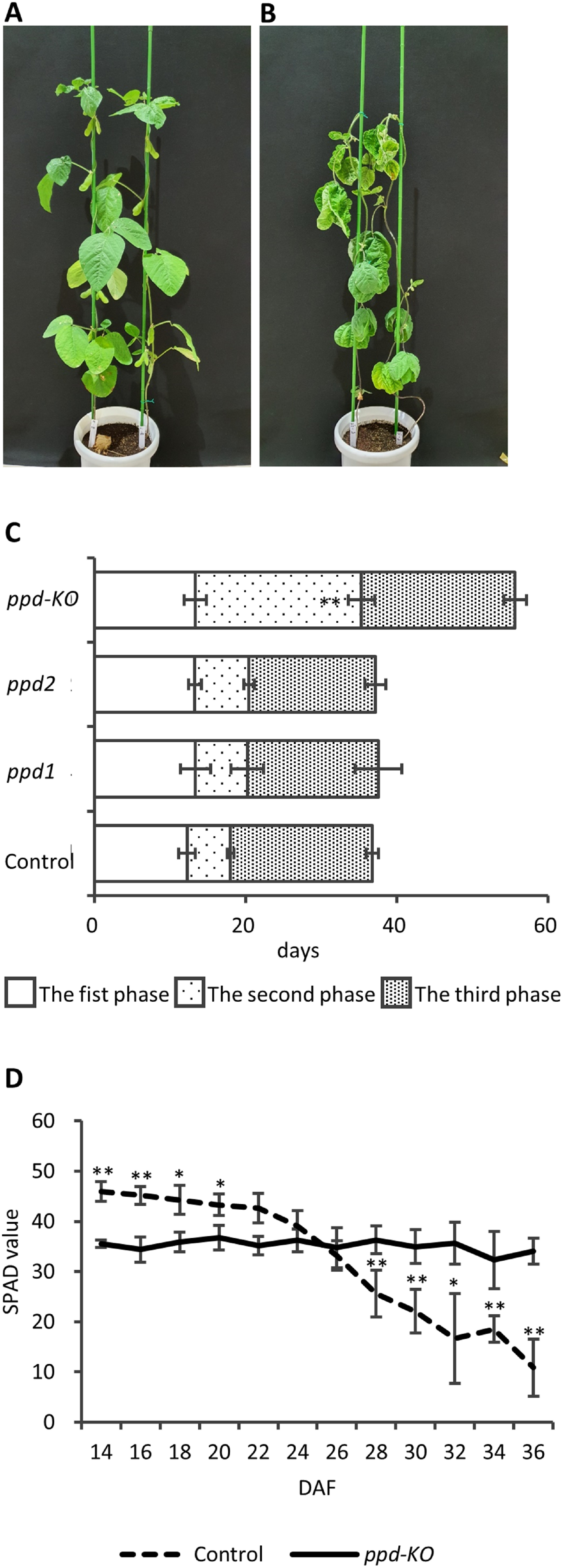
Figure 3. External morphological and physiological characteristics of control plants and the mutants. External morphological character of control plants (A) and the *ppd-KO* mutant (B) 63 days after flowering. (C) Growing periods of the 1 cm-sized pods from sprouting to development of control plants and the mutants. ** indicates significant differences between control plants and the mutants at the 1% level. All data shown are the mean±SE of 3–10 plants. (D) SPAD value of the first trifoliolate leaves of control plants and the *ppd-KO* mutant from 14 days after flowering. All data were collected as four area of one trifoliolate region of three plants each. All data shown are mean±SE. * and ** indicate significant differences between control plants and the mutants at the 5% and 1% levels, respectively.

The growth durations of control plants and the *ppd-KO* mutant were considerably different. The *ppd-KO* mutant did not develop pods by the time the control plants did (Figure 3A, B). The first growth phase was not different between the mutants and control plants (Figure 3C). The first growth phase lasted approximately 6 days (Figure 3C). However, the second growth phase of the *ppd-KO* mutant was approximately 14 days longer than the other mutants and control plants (Figure 3C). This resulted in delayed flowering time in the *ppd-KO* mutant compared with that in the other mutants and control plants. Nevertheless, there was no difference in the third growth phase among the mutants, and the phase lasted approximately 18 days (Figure 3C). The *ppd-KO* mutant exhibited leaf senescence characteristics different from those of the other mutants and control plants. The control plants showed a decrease in SPAD values 22 DAF (Figure 3D). However, the *ppd1* and *ppd2* mutants showed the same leaf senescence pattern (data not shown). The SPAD value of the *ppd-KO* mutant was slightly lower than that of control plants; however, the SPAD value remained unchanged after 36 DAF. Further, the *ppd-KO* mutant exhibited a stay-green phenotype (Figure 3D).

### Numbers of guard cells on the unifoliolate leaves of the mutants

**Figure 4:**
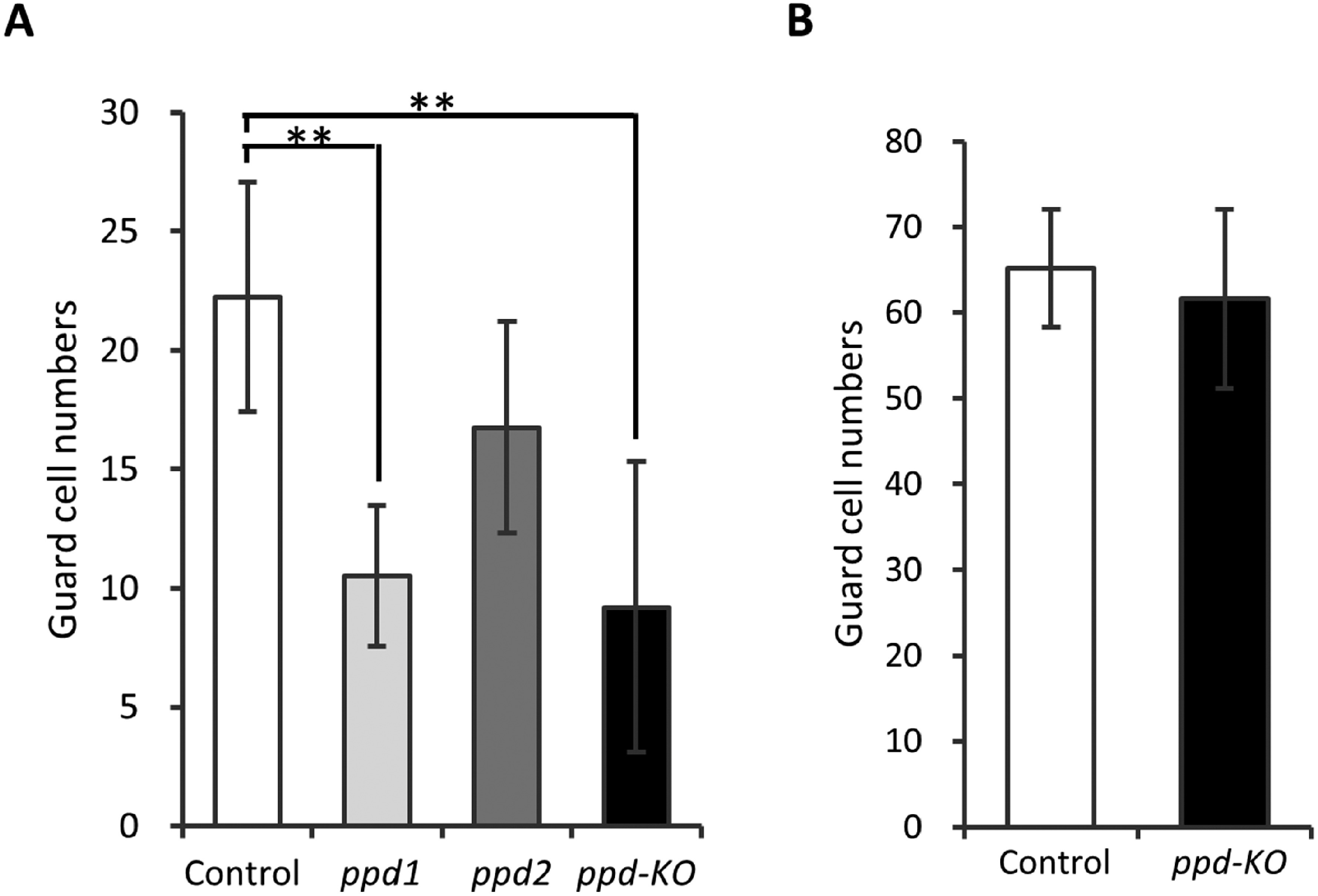
Figure 4. Guard cell numbers in the unifoliolate leaves of control plants and the *ppd-KO* mutant. (A) The adaxial side of the unifoliolate leaves. (B) The abaxial side of the unifoliolate leaves. All data were collected from every five areas (0.6 mm^2^) of one the unifoliolate leaf in five plants. All data shown are mean±SE. ** indicates significant differences between control plants and the mutants at the 1% level.

We determined the number of guard cells on the abaxial and adaxial sides of the unifoliolate leaves of the mutants and control plants. The number of guard cells was 22 cells/0.6 mm^2^ on the adaxial side of the unifoliolate leaves of control plants (Figure 4A) and was considerably higher than that on the adaxial side of the unifoliolate leaves of the *ppd1* and *ppd-KO* mutants (Figure 4A). The abaxial side of the unifoliolate leaves of the *ppd-KO* mutant, which had the lowest number of guard cells on the adaxial side, was compared with that of control plants. No difference was observed in the number of guard cells on the abaxial side of the unifoliolate leaves (Figure 4B).

### Gene expression analysis of the *ppd-KO* mutant and control plants

**Figure 5:**
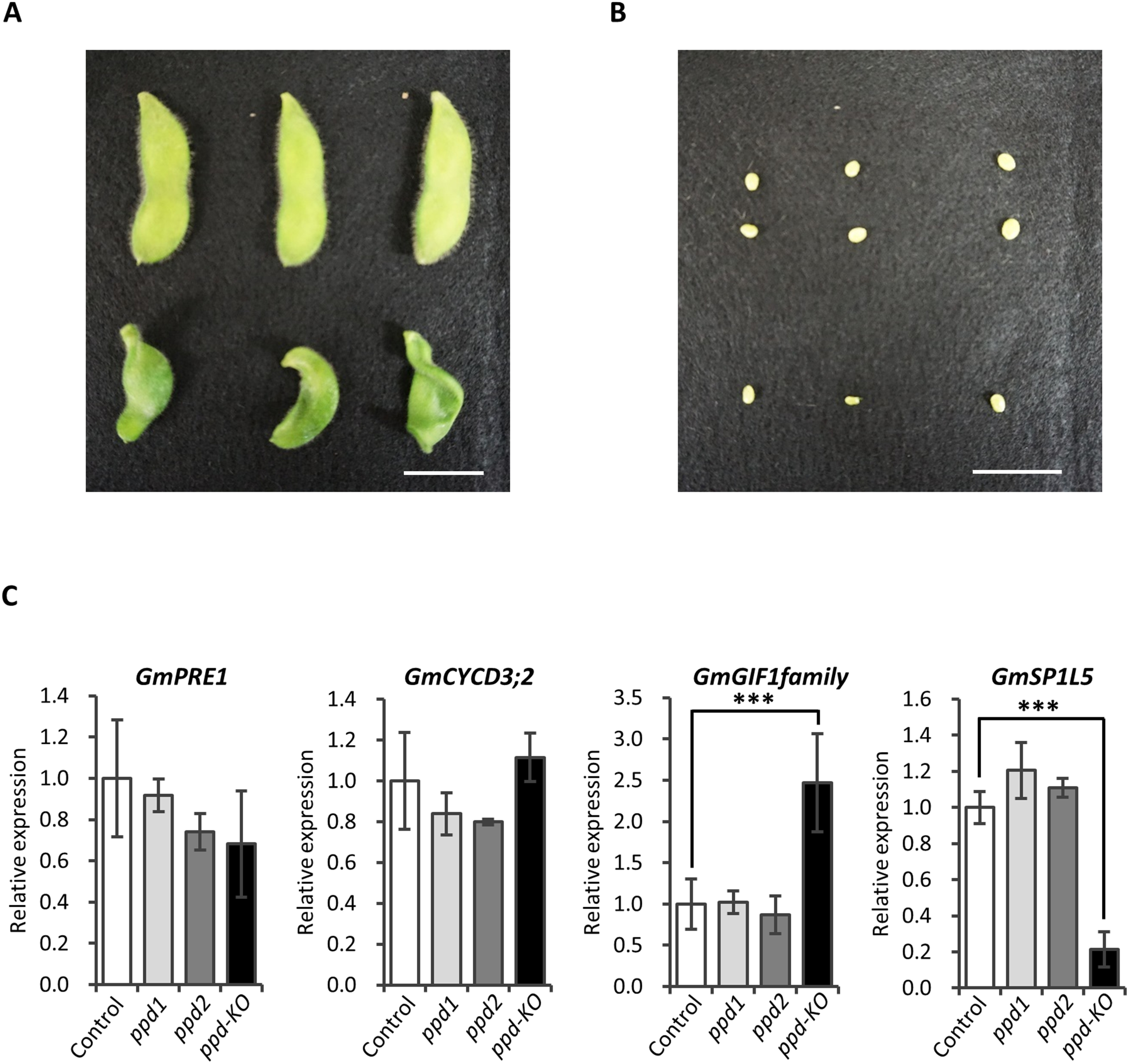
Figure 5. Morphological characteristics of the pods and gene expression analysis of control plants and the *ppd-KO* mutant. (A) Pods 21 days after flowering. Upside, Control plant; bottom side, *ppd-KO* mutant. (B) Embryos 21 days after flowering. Upside, Control plant; bottom side, *ppd-KO* mutant. (C) Gene expression analysis of 1 cm-sized pods of control plants and the *ppd* mutants. The expression of each gene was normalized to that of *Bic-C2* (Glyma.03G064800). *** indicates significant differences between control plants and the *ppd* mutants at the 0.1% levels. All data shown are the mean±SE of 3–4 biological replicates.

To investigate why the *ppd-KO* mutant produced fewer seeds, the shape of the young pods and development of embryos were observed in the *ppd-KO* mutant and control plants 21 DAF. The *ppd-KO* mutant exhibited a prominently twisted pod phenotype (Figure 5A). However, developed embryos were found in these twisted pods and control plants (Figure 5B). Subsequently, transcriptome analysis was performed on 1 cm-sized pods of the *ppd-KO* mutant and control plants, which were collected 7 DAF. In total, 128 differentially expressed genes were identified between the *ppd-KO* mutant and control plants (Supplementary Table S2). The genes involved in cell division and organ enlargement were SPIRAL1-LIKE 5 (*GmSP1L5*; Glyma.08G017200), *GmGIF1* (Glyma.03G249000; Glyma.19G246600; Glyma.20G226500; and Glyma.10G164100), and PACLOBUTRAZOL RESISTANCE 1 (*GmPRE1*; Glyma.18G258700). Therefore, qRT-PCR analysis was performed using 1 cm-sized pods of the mutants and control plants to analyze the expression of the abovementioned genes and *GmCYCD3;2* (Glyma.17G167700), which is reported to be regulated by PPD. The expression of GIF1 family genes was noticeably higher in 1 cm-sized pod of the *ppd-KO* mutant than in that of control plants (Figure 5C). In addition, the expression of *GmSP1L5* was considerably lower in 1 cm-sized pods of the *ppd-KO* mutant than in that of control plants (Figure 5C). These changes in gene expression level were also observed in the unifoliolate leaves of the *ppd-KO* mutant (Supplementary Figure S2). In contrast, there were no significant differences in these genes between the *ppd1* or *ppd2* mutants and the control plants (Figure 5C).

## Discussion

In this study, *ppd1* and *ppd2* mutants exhibited a phenotype of organ enlargement (Figures 1A, 2A) similar to that of *GmPPD-*knocked down plants (Ge et al. 2016; Naito et al. 2017). The *ppd1* and *ppd2* mutants lacked 15 and 13 amino acid residues at the *GmPPD2* and *GmPPD1* loci, respectively, owing to in-frame mutations (Supplementary Figure S1). These deleted regions were located away from the PPD and TIFY domains (Bai et al. 2011; Cuéllar Pérez et al. 2014), which play important roles as transcription factors in PPD. Our findings suggest that the *ppd1* and *ppd2* mutants partially retain the function of the PPD protein. In contrast, the *ppd-KO* mutant, which harbored frameshift mutations at both the *GmPPD1* and *GmPPD2* loci, exhibited drastic phenotype differences, such as wrinkled and dome-shaped leaves and twisted pods (Figures 3B, 5A). These observations indicate that partial retention of the PPD function would be essential for tissue enlargement in soybean.

Although the *ppd-KO* mutant rarely produced seeds, immature embryos were formed in young pods 21 DAF (Figures 1A, 1B, 5B). These pods exhibited a twist phenotype (Figure 5A). These findings indicate that pod twisting may physically inhibit embryo development. Collectively, the findings suggest that complete loss of PPD function results in considerably impairment in soybean organogenesis. The *ppd-KO* mutant exhibited an extended growth phase from the development of the unifoliolate leaves to that of the first trifoliolate leave (Figure 3), which was about 2 weeks longer than that of control plant (Figure 3). The *ppd-KO* mutant also exhibited a stay-green phenotype that suppressed chlorophyll degradation during plant senescence (Figure 3D). This stay-green phenotype has not been previously reported in other *PPD* mutants. Studies have reported two types of spontaneous stay-green mutants in soybeans, and the genes responsible for this phenotype have been identified (Fang et al. 2014; Kohzuma et al. 2017; Nakano et al. 2014). However, these genes were not present among the differentially expressed genes identified via transcriptome analysis in the present study. Further, the *ppd-KO* mutant maintained this stay-green phenotype until the plant body died (data not shown). Collectively, loss of PPD function in soybean affects transition of developmental stage and photosynthetic pigment biosynthesis and metabolism. These findings may provide novel insights into the PPD function in higher plants.

In Arabidopsis, GIFs are transcriptional cofactors that regulate plant growth and development (Debernardi et al. 2014; Kim and Kende 2004). The *gif1* mutant of Arabidopsis exhibited decreased leaf and petal sizes (Lee et al. 2009). *G1F1* expression is controlled by the protein complex containing PPD (Liu et al. 2020). Knockdown of *GmPPD* via RNAi also increased the expression of *GIF1* and the seed weight and leaf size of transgenic soybean plants (Ge et al. 2016). The expression of *GIF1* family genes was significantly increased in 1 cm-sized pods of the *ppd-KO* mutant and the unifoliolate leaves of the *ppd-KO* mutant (Figure 5C, Supplementary Figure S2). However, unlike PPD-knocked down plants, the *ppd-KO* mutant did not exhibit the organ enlargement phenotype but exhibited a twisted pod phenotype and produced fewer seeds. It was not possible to determine whether increased expression of the *GmGIF1* family genes is essential for the phenotype of the *ppd-KO* mutant, but the production of transgenic soybean plants overexpression the *GmGIF1* gene may reveal the relationship between the expression level of *GIF1* gene and the twisted pod phenotype. Gene expression analysis also revealed considerable downregulation of *GmSP1L5* in the 1 cm-sized pods of the *ppd-KO* mutant (Figure 5C). In Arabidopsis, SPIRAL1 (SPR1) and SP1L act redundantly to maintain the cortical microtubule organization essential for anisotropic cell growth (Furutani et al. 2000; Nakajima et al. 2006). Multiple-mutant *spr1* and *sp1l* plants exhibited right-handed tendril-like twinning growth (Nakajima et al. 2006). These findings support the hypothesis that considerable downregulation of *SP1L5* results in twisted pods in the *ppd-KO* mutant. Of date, there are no reports on the relationship between PPD and SP1L5. We revealed that loss of PPD function affects the expression of *SP1L5* in soybean.

In this study, we demonstrated that while the decrease in PPD function contributed to organ enlargement, complete knockout of *PPD* had a negative effect on soybean organogenesis. Loss of PPD function also delayed vegetative growth and inhibited chlorophyll metabolism in soybean plants. This information could be useful for improving crop yield and growing time.

## Abbreviations

DAF: days after flowering
qRT-PCR: quantitative RT-PCR
RNA-seq: RNA sequencing

## Description of Supplementary Files

Supplementary Table S1. Primer sequences used for genotyping, sequencing, and gene expression analysis

Supplementary Table S2. Changes in gene expression identified in *ppd-KO* and control plants using RNA-seq

Supplementary Figure S1. Targeted region of the gRNA and nucleotide and predicted amino acid sequences of the targeted and flanking regions

Supplementary Figure S2. Gene expression analysis of 1 cm-sized pods of the control and *ppd1* and *ppd2* mutant plants
