# Supplementary material for: Site-directed mutagenesis of soybean PEAPOD genes using the CRISPR/Cas9 system alters tissue developmental transition
Source: Plant Biotechnol (Tokyo). 2023 Sep 25;40(3):247–54. doi: 10.5511/plantbiotechnology.23.0628a (PMC10901156; doi:10.5511/plantbiotechnology.23.0628a)
Supplement: Supplementary Data [file plantbiotechnology-40-3-23.0628a-s001.pdf]

Supplementary Table S1. Primer sequences used for genotyping, sequencing, and gene expression analyses

Primer name	Primer sequence (5'-3')	Use of amplicon
PRE1 Fw	GTGCCTCTTTTTGTGGATGG	RT-qPCR analyses
PRE1 Rv	CTTCCAACCTCTACGTGACTG	RT-qPCR analyses
SPIRAL1 08G Fw	AAGAACAGCCAAAGCCTCCT	RT-qPCR analyses
SPIRAL1 08G Rv	GTTGGCCTTGACCAGTAGGG	RT-qPCR analyses
GIF1-03Gq Fw	CACACATGCACTTCTCTTCTTC	RT-qPCR analyses
GIF1-03Gq Rv	CAAAGCAAATTGGTGAGGAGAA	RT-qPCR analyses
GIF1-19Gq Fw	CTCAGCTTTGTTTGGTTTTGGA	RT-qPCR analyses
GIF1-19Gq Rv	CCATCATGGGCTGCATCTG	RT-qPCR analyses
GIF1-20Gq Fw	TCTCTCCCTGTGTTTGTCCA	RT-qPCR analyses
GIF1-20Gq Rv	CTGTTTTCTCTCTGGTCCCG	RT-qPCR analyses
GIF1-10Gq Fw	GCAAACTGCCTTAACGTTTCT	RT-qPCR analyses
GIF1-10Gq Rv	GCCAACAAGTGTGCATACAG	RT-qPCR analyses
CYCD3;2 17G Fw	ATGGTGTCGTTGCTAATGGG	RT-qPCR analyses
CYCD3;2 17G Rv	CCAGTTCACAGCCTCAACAC	RT-qPCR analyses
ACTIN2/7 Fw	ATTCACGAGACCACCTACAAC	Control Gene for qPCR analyses
ACTIN2/7 Rv	TGAGCCACCACTAAGAACAATG	Control Gene for qPCR analyses
Bic-C2 Fw	GCTATCAGGGACAATGGCAC	Control Gene for qPCR analyses
Bic-C2 Rv	CCATGACTTTTTAGGAGCTGGG	Control Gene for qPCR analyses
ELF1b.B Fw	TGGCTGTCACCTTCTCAGAT	Control Gene for qPCR analyses
ELF1b.B Rv	GATCGAGGGATTTGATGCCC	Control Gene for qPCR analyses
PPD20G-L2	ACCACCGTAGTCATGCTCCTC	CAPS and sequencing analyses
PPD20G-R2	TCCGTATCATCTCCCACCTCAC	CAPS and sequencing analyses
PPD10G-L	CACCGTAGCCATGCTCAAC	CAPS and sequencing analyses
PPD10G-R	AACCTTAACGTTTTCAAGTTTAACC	CAPS and sequencing analyses

Supplementary Table S2. Expression fluctuation genes identified between the *ppd-KO* and control plants by RNA-seq analysis

Gene	Defline	sample_1	sample_2	value_1	value_2	log2(fold_change)	test_stat	p_value	q_value
Glyma.01G115100	(1 of 1) PTHR10639:SF10 - CLATHRIN LIGHT CHAIN 2	control	*ppd-KO*	0	3.21016	inf	NaN	5.00E-05	0.0157425
Glyma.01G142400	(1 of 4) PTHR11177//PTHR11177:SF190 - CHITINASE	control	*ppd-KO*	109.98	18.1714	-2.5975	-5.58652	5.00E-05	0.0157425
Glyma.01G151100	(1 of 2306) 2.7.11.1 - Non-specific serine/threonine protein kinase / Threonine-specific protein kinase	control	*ppd-KO*	0	0.962508	inf	NaN	5.00E-05	0.0157425
Glyma.02G017500	(1 of 4) 4.1.99.13 - (6-4)DNA photolyase / DNA photolyase	control	*ppd-KO*	4.22902	42.7975	3.33913	6.42653	5.00E-05	0.0157425
Glyma.02G093200	(1 of 8) PTHR19375:SF232 - MEDIATOR OF RNA POLYMERASE II TRANSCRIPTION SUBUNIT 37E-RELATED	control	*ppd-KO*	29.2717	1.49517	-4.29112	-7.49859	5.00E-05	0.0157425
Glyma.02G099200	(1 of 6) PF06351 - Allene oxide cyclase (Allene_ox_cyc)	control	*ppd-KO*	49.6341	7.20681	-2.7839	-5.34815	5.00E-05	0.0157425
Glyma.02G132100	(1 of 2) PTHR32439:SF0 - FERREDOXIN--NITRITE REDUCTASE, CHLOROPLASTIC	control	*ppd-KO*	15.5291	3.83552	-2.01748	-4.143	5.00E-05	0.0157425
Glyma.02G213000	(1 of 4) PTHR13683:SF259 - ASPARTYL PROTEASE-LIKE PROTEIN	control	*ppd-KO*	34.0091	4.2858	-2.98828	-5.75625	5.00E-05	0.0157425
Glyma.02G245600	(1 of 6) PTHR23201:SF1 - GIBBERELLIN-REGULATED PROTEIN 12-RELATED	control	*ppd-KO*	116.287	15.0756	-2.9474	-5.92138	5.00E-05	0.0157425
Glyma.03G010200	(1 of 20) PF08372 - Plant phosphoribosyltransferase C-terminal (PRT_C)	control	*ppd-KO*	4.0453	0	-inf	NaN	5.00E-05	0.0157425
Glyma.03G027100		control	*ppd-KO*	0	0.792407	inf	NaN	5.00E-05	0.0157425

Glyma.03G088000	(1 of 58) PTHR11017//PTHR11017:SF163 - LEUCINE-RICH REPEAT-CONTAINING PROTEIN	control	*ppd-KO*	1.32546	0	-inf	NaN	5.00E-05	0.0157425
Glyma.03G088100	(1 of 58) PTHR11017//PTHR11017:SF163 - LEUCINE-RICH REPEAT-CONTAINING PROTEIN	control	*ppd-KO*	1.8487	0	-inf	NaN	5.00E-05	0.0157425
Glyma.03G088200	(1 of 13) PF14683 - Polysaccharide lyase family 4, domain III (CBM-like)	control	*ppd-KO*	1.79042	0	-inf	NaN	5.00E-05	0.0157425
Glyma.03G091700	(1 of 9) PTHR11064//PTHR11064:SF45 - CCAAT-BINDING TRANSCRIPTION FACTOR-RELATED	control	*ppd-KO*	0	1.26198	inf	NaN	5.00E-05	0.0157425
Glyma.03G098600	(1 of 7) 1.1.1.54//1.3.1.102//1.3.1.74 - 2-alkenal reductase (NADP(+)) / NADPH-dependent alkenal/one oxidoreductase // 2-alkenal reductase (NAD(P)(+)) / NADPH:2-alkenal alpha,beta-hydrogenase	control	*ppd-KO*	1.58093	50.1596	4.98768	7.65994	5.00E-05	0.0157425
Glyma.03G109600	(1 of 6) PTHR22940 - TIMEOUT/TIMELESS-2	control	*ppd-KO*	2.36665	0	-inf	NaN	5.00E-05	0.0157425
Glyma.03G132700	(1 of 5) PTHR32227:SF49 - BETA-1,3-GLUCANASE 1-RELATED	control	*ppd-KO*	42.5448	8.76423	-2.27928	-4.79662	5.00E-05	0.0157425
Glyma.03G222600	(1 of 3) 1.1.1.145 - 3-beta-hydroxy-Delta(5)-steroid dehydrogenase / Progesterone reductase	control	*ppd-KO*	0	4.99659	inf	NaN	5.00E-05	0.0157425
Glyma.03G223600	(1 of 7) PTHR24078:SF255 - DNAJ HOMOLOG DNJ-5	control	*ppd-KO*	7.43783	27.6835	1.89607	4.08379	5.00E-05	0.0157425
Glyma.03G249000	(1 of 7) PTHR23107:SF6 - GRF1-INTERACTING FACTOR 1	control	*ppd-KO*	9.10661	50.589	2.47384	5.18718	5.00E-05	0.0157425
Glyma.03G252600	(1 of 3) PTHR22835//PTHR22835:SF171 - ZINC FINGER FYVE DOMAIN CONTAINING PROTEIN	control	*ppd-KO*	2.11551	0	-inf	NaN	5.00E-05	0.0157425
Glyma.04G104700	(1 of 3) PTHR22753:SF1 - TRANSMEMBRANE PROTEIN 68	control	*ppd-KO*	3.63111	0	-inf	NaN	5.00E-05	0.0157425

Glyma.04G114500		control	*ppd-KO*	0	5.37736	inf	NaN	5.00E-05	0.0157425
Glyma.04G114600		control	*ppd-KO*	0	5.37736	inf	NaN	5.00E-05	0.0157425
Glyma.04G114702		control	*ppd-KO*	0	5.37736	inf	NaN	5.00E-05	0.0157425
Glyma.04G179500	(1 of 7) PTHR23201//PTHR23201:SF18 - EXTENSIN, PROLINE-RICH PROTEIN	control	*ppd-KO*	11.0045	165.189	3.90794	7.23104	5.00E-05	0.0157425
Glyma.04G180300	(1 of 2306) 2.7.11.1 - Non-specific serine/threonine protein kinase / Threonine-specific protein kinase	control	*ppd-KO*	35.1678	0	-inf	NaN	5.00E-05	0.0157425
Glyma.05G002500	(1 of 1) PTHR10457:SF13 - GLUCURONOKINASE 1-RELATED	control	*ppd-KO*	5.50398	34.9362	2.66617	5.39619	5.00E-05	0.0157425
Glyma.05G036800	(1 of 5) PTHR13935//PTHR13935:SF59 - ACHAETE-SCUTE TRANSCRIPTION FACTOR-RELATED	control	*ppd-KO*	0	2.65744	inf	NaN	5.00E-05	0.0157425
Glyma.05G050700	(1 of 2) PTHR11945:SF191 - AGAMOUS-LIKE MADS-BOX PROTEIN AGL14-RELATED	control	*ppd-KO*	0.629228	0	-inf	NaN	5.00E-05	0.0157425
Glyma.05G057000	(1 of 2) PTHR31731:SF8 - BIFUNCTIONAL INHIBITOR/LIPID-TRANSFER PROTEIN/SEED STORAGE 2S ALBUMIN SUPERFAMILY PROTEIN	control	*ppd-KO*	107.576	25.1906	-2.09439	-4.54989	5.00E-05	0.0157425
Glyma.05G057200	(1 of 36) PF14547 - Hydrophobic seed protein (Hydrophob_seed)	control	*ppd-KO*	57.6209	5.523	-3.38307	-5.69506	5.00E-05	0.0157425
Glyma.05G149900	(1 of 3) PTHR10516:SF324 - PEPTIDYL-PROLYL CIS-TRANS ISOMERASE FKBP62	control	*ppd-KO*	13.1042	3.07415	-2.09177	-4.26154	5.00E-05	0.0157425
Glyma.05G154600	(1 of 5) PTHR33044:SF28 - BIFUNCTIONAL INHIBITOR/LIPID-TRANSFER PROTEIN/SEED STORAGE 2S ALBUMIN SUPERFAMILY PROTEIN	control	*ppd-KO*	99.9056	25.4516	-1.97281	-4.30802	5.00E-05	0.0157425
Glyma.05G222000	(1 of 2) PTHR10457:SF11 - GALACTURONOKINASE	control	*ppd-KO*	10.2233	86.28	3.07716	6.45477	5.00E-05	0.0157425

Glyma.05G224500	(1 of 7) 1.13.99.1 - Inositol oxygenase / Myo-inositol oxygenase	control	*ppd-KO*	4.91807	113.609	4.52984	8.61652	5.00E-05	0.0157425
Glyma.05G236500	(1 of 27) PF01985 - CRS1 / YhbY (CRM) domain (CRS1_YhbY)	control	*ppd-KO*	1.32714	0	-inf	NaN	5.00E-05	0.0157425
Glyma.06G024500	(1 of 5) PTHR23201:SF2 - GIBBERELLIN-REGULATED PROTEIN 1-RELATED	control	*ppd-KO*	0	3.15518	inf	NaN	5.00E-05	0.0157425
Glyma.06G100300	(1 of 91) PF02298 - Plastocyanin-like domain (Cu_bind_like)	control	*ppd-KO*	228.731	34.2691	-2.73867	-5.88265	5.00E-05	0.0157425
Glyma.06G121100	(1 of 5) PTHR33021:SF9 - BASIC BLUE PROTEIN	control	*ppd-KO*	112.795	31.0014	-1.8633	-4.07745	5.00E-05	0.0157425
Glyma.06G302200	(1 of 2) 4.2.3.106 - (E)-beta-ocimene synthase / Beta-ocimene synthase	control	*ppd-KO*	0	0.489486	inf	NaN	5.00E-05	0.0157425
Glyma.07G050300	(1 of 8) 3.1.30.1 - Aspergillus nuclease S(1) / Single-stranded-nucleate endonuclease	control	*ppd-KO*	26.2257	101.795	1.95661	4.26755	5.00E-05	0.0157425
Glyma.07G051000	(1 of 5) PTHR24343//PTHR24343:SF199 - SERINE/THREONINE KINASE	control	*ppd-KO*	9.2037	31.7998	1.78873	3.88197	0.00015	0.0420621
Glyma.07G126200	(1 of 4) 2.3.2.15//2.3.2.2//3.4.19.13 - Glutathione gamma-glutamylcysteinyltransferase / Phytochelatin synthase // Gamma-glutamyltransferase / Glutamyl transpeptidase // Glutathione hydrolase / Glutathionase	control	*ppd-KO*	5.82194	18.981	1.70498	3.70113	0.0001	0.0296636
Glyma.07G133500	(1 of 19) 3.1.3.74 - Pyridoxal phosphatase / Vitamin B6-phosphate phosphatase	control	*ppd-KO*	77.168	398.001	2.3667	4.40325	0.00015	0.0420621
Glyma.07G150900	(1 of 10) 1.14.11.23 - Flavonol synthase / FLS	control	*ppd-KO*	3.35988	57.8161	4.10499	7.34719	5.00E-05	0.0157425
Glyma.07G187600	(1 of 4) 4.2.3.48 - (3S,6E)-nerolidol synthase / Nerolidol synthase	control	*ppd-KO*	2.14044	13.9897	2.70839	4.73622	5.00E-05	0.0157425
Glyma.07G198800	(1 of 79) PF02701 - Dof domain, zinc finger (zf-Dof)	control	*ppd-KO*	0	2.99214	inf	NaN	5.00E-05	0.0157425

Glyma.07G223500	(1 of 8) PTHR31375:SF2 - POLYGALACTURONASE/PECTINASE	control	*ppd-KO*	1.77845	0	-inf	NaN	5.00E-05	0.0157425
Glyma.08G017200	(1 of 4) PTHR33403:SF2 - PROTEIN SPIRAL1-LIKE 5	control	*ppd-KO*	549.161	108.714	-2.33669	-4.72638	5.00E-05	0.0157425
Glyma.08G071300	(1 of 4) PTHR31279:SF12 - PHI-1-LIKE PROTEIN	control	*ppd-KO*	5.63109	21.8166	1.95394	4.08817	5.00E-05	0.0157425
Glyma.08G127600	(1 of 44) PF00190 - Cupin (Cupin_1)	control	*ppd-KO*	21.405	2.76692	-2.9516	-5.1619	5.00E-05	0.0157425
Glyma.08G155700	(1 of 252) PF02519 - Auxin responsive protein (Auxin_inducible)	control	*ppd-KO*	0	1.64063	inf	NaN	5.00E-05	0.0157425
Glyma.08G158200		control	*ppd-KO*	1.2865	0	-inf	NaN	5.00E-05	0.0157425
Glyma.08G189200	(1 of 35) 1.13.11.58 - Linoleate 9S-lipoxygenase / Linoleate 9-lipoxygenase	control	*ppd-KO*	1.88746	10.1319	2.42438	4.55899	5.00E-05	0.0157425
Glyma.08G318400	(1 of 6) PTHR22835//PTHR22835:SF116 - ZINC FINGER FYVE DOMAIN CONTAINING PROTEIN	control	*ppd-KO*	3.30552	28.044	3.08474	5.50785	5.00E-05	0.0157425
Glyma.08G321400	(1 of 10) PTHR13683:SF246 - ASPARTYL PROTEASE FAMILY PROTEIN	control	*ppd-KO*	20.0513	154.603	2.9468	6.06377	5.00E-05	0.0157425
Glyma.08G342000	(1 of 22) PTHR33107:SF5 - KUNITZ FAMILY TRYPSIN AND PROTEASE INHIBITOR PROTEIN-RELATED	control	*ppd-KO*	410.196	46.3128	-3.14683	-6.25406	5.00E-05	0.0157425
Glyma.09G004400	(1 of 3) 1.8.4.10 - Adenylyl-sulfate reductase (thioredoxin) / Thioredoxin-dependent 5'-adenylylsulfate reductase	control	*ppd-KO*	46.0174	11.9541	-1.94467	-4.28329	5.00E-05	0.0157425
Glyma.09G010800	(1 of 3) PTHR10283:SF82 - SOLUTE CARRIER FAMILY 13 MEMBER 5	control	*ppd-KO*	3.03265	0	-inf	NaN	5.00E-05	0.0157425
Glyma.09G031600	(1 of 7) PF13229 - Right handed beta helix region (Beta_helix)	control	*ppd-KO*	9.74748	1.1562	-3.07564	-4.81715	0.0001	0.0296636
Glyma.09G048400	(1 of 2) PTHR31235:SF8 - PEROXIDASE 7	control	*ppd-KO*	0	1.55329	inf	NaN	5.00E-05	0.0157425
Glyma.09G057500	(1 of 2) KOG0296 - Angio-associated migratory cell protein (contains WD40 repeats)	control	*ppd-KO*	0	1.52964	inf	NaN	5.00E-05	0.0157425

Glyma.09G075200	(1 of 20) 2.3.1.74 - Naringenin-chalcone synthase / Flavonone synthase	control	*ppd-KO*	0	0.713853	inf	NaN	5.00E-05	0.0157425
Glyma.09G077600	(1 of 16) PTHR12899 - 39S RIBOSOMAL PROTEIN L18, MITOCHONDRIAL	control	*ppd-KO*	63.2232	2.04841	-4.94788	-7.28831	5.00E-05	0.0157425
Glyma.09G092700	(1 of 1) PTHR32093:SF16 - GENOMIC DNA, CHROMOSOME 3, P1 CLONE:MKA23-RELATED	control	*ppd-KO*	114.583	21.9947	-2.38117	-4.79439	5.00E-05	0.0157425
Glyma.09G107100	(1 of 6) PTHR10209:SF134 - 2-OXOGLUTARATE (2OG) AND FE(II)-DEPENDENT OXYGENASE-LIKE PROTEIN	control	*ppd-KO*	4.8457	47.5589	3.29494	5.53645	5.00E-05	0.0157425
Glyma.09G152100	(1 of 2) KOG3461 - CDGSH-type Zn-finger containing protein	control	*ppd-KO*	127.182	32.2823	-1.97808	-4.33159	5.00E-05	0.0157425
Glyma.09G163900	(1 of 50) PF00197 - Trypsin and protease inhibitor (Kunitz_legume)	control	*ppd-KO*	5.48141	48.2176	3.13694	5.75375	5.00E-05	0.0157425
Glyma.09G184300	(1 of 1) PF00170//PF00464 - bZIP transcription factor (bZIP_1) // Serine hydroxymethyltransferase (SHMT)	control	*ppd-KO*	3.64769	0	-inf	NaN	5.00E-05	0.0157425
Glyma.09G198900	(1 of 2) PTHR21495//PTHR21495:SF60 - NUCLEOPORIN-RELATED	control	*ppd-KO*	4.44872	47.8879	3.4282	6.05516	5.00E-05	0.0157425
Glyma.11G106300	(1 of 6) PTHR21576:SF17 - MAJOR FACILITATOR FAMILY PROTEIN	control	*ppd-KO*	23.6057	94.0194	1.99382	4.15913	5.00E-05	0.0157425
Glyma.11G120400	(1 of 113) PF14368 - Probable lipid transfer (LTP_2)	control	*ppd-KO*	241.434	51.7961	-2.22071	-4.78995	5.00E-05	0.0157425
Glyma.11G141700	(1 of 16) PTHR23428 - HISTONE H2B	control	*ppd-KO*	284.648	49.0226	-2.53766	-5.2206	5.00E-05	0.0157425
Glyma.11G153200	(1 of 5) PTHR22979 - ZINC FINGER PROTEIN-RELATED	control	*ppd-KO*	17.8358	0	-inf	NaN	5.00E-05	0.0157425
Glyma.11G221000	(1 of 9) KOG0651 - 26S proteasome regulatory complex, ATPase RPT4	control	*ppd-KO*	205.159	35.7401	-2.52113	-5.03064	5.00E-05	0.0157425
Glyma.12G013100	(1 of 2) PTHR11527:SF95 - 23.5 KDA HEAT SHOCK PROTEIN, MITOCHONDRIAL-RELATED	control	*ppd-KO*	55.7528	6.30982	-3.14337	-5.97881	5.00E-05	0.0157425

Glyma.12G015100	(1 of 4) PTHR11695//PTHR11695:SF568 - ALCOHOL DEHYDROGENASE RELATED	control	*ppd-KO*	2.19157	15.0838	2.78296	4.66227	0.0001	0.0296636
Glyma.12G076700	(1 of 4) PTHR31223:SF23 - CYTOKININ RIBOSIDE 5'-MONOPHOSPHATE PHOSPHORIBOHYDROLASE LOG1	control	*ppd-KO*	22.3977	79.2637	1.82331	4.00965	0.00015	0.0420621
Glyma.12G093000		control	*ppd-KO*	34.3989	5.19031	-2.72847	-4.71681	5.00E-05	0.0157425
Glyma.13G043500	(1 of 1) PF00005//PF00076//PF01061 - ABC transporter (ABC_tran) // RNA recognition motif. (a.k.a. RRM, RBD, or RNP domain) (RRM_1) // ABC-2 type transporter (ABC2_membrane)	control	*ppd-KO*	0	0.439336	inf	NaN	5.00E-05	0.0157425
Glyma.13G094200	(1 of 3) PTHR10334:SF204 - ALLERGEN V5/TPX-1-RELATED FAMILY PROTEIN-RELATED	control	*ppd-KO*	21.3131	4.23333	-2.33188	-4.2984	0.0001	0.0296636
Glyma.13G109800	(1 of 4) PTHR22893:SF67 - 12-OXOPHYTODIENOATE REDUCTASE 3	control	*ppd-KO*	0	2.70955	inf	NaN	5.00E-05	0.0157425
Glyma.13G183500	(1 of 3) 1.10.3.1//1.14.18.1 - Catechol oxidase / Tyrosinase // Tyrosinase / Tyrosine-dopa oxidase	control	*ppd-KO*	6.10512	43.2604	2.82495	5.93028	5.00E-05	0.0157425
Glyma.13G213300	(1 of 12) PTHR23500:SF62 - SUGAR TRANSPORTER ERD6-LIKE 14-RELATED	control	*ppd-KO*	0	0.900017	inf	NaN	5.00E-05	0.0157425
Glyma.13G257500		control	*ppd-KO*	2.52828	0	-inf	NaN	5.00E-05	0.0157425
Glyma.13G285200	(1 of 4) 4.2.3.22 - Germacradienol synthase / Germacradienol/germacrene-D synthase	control	*ppd-KO*	0	1.37473	inf	NaN	5.00E-05	0.0157425
Glyma.14G010500	(1 of 2) PTHR31268:SF4 - GALACTINOL--SUCROSE GALACTOSYLTRANSFERASE 1-RELATED	control	*ppd-KO*	6.12743	36.8241	2.5873	5.55329	5.00E-05	0.0157425

Glyma.14G038300	(1 of 2) PTHR11413//PTHR11413:SF57 - CYSTATIN FAMILY MEMBER	control	*ppd-KO*	56.2113	267.099	2.24844	4.75292	5.00E-05	0.0157425
Glyma.14G156400	(1 of 2) PTHR11695//PTHR11695:SF493 - ALCOHOL DEHYDROGENASE RELATED	control	*ppd-KO*	61.1417	16.6303	-1.87835	-4.13272	0.0001	0.0296636
Glyma.14G162900		control	*ppd-KO*	1.72744	0	-inf	NaN	5.00E-05	0.0157425
Glyma.14G187300	(1 of 3) PTHR31851:SF16 - MEMBRANE PROTEIN OF ER BODY 2	control	*ppd-KO*	7.4317	25.9958	1.80651	3.96718	0.0001	0.0296636
Glyma.14G209400	(1 of 4) 1.5.3.16//1.5.3.17 - Spermine oxidase / SMO // Non-specific polyamine oxidase / Polyamine oxidase	control	*ppd-KO*	1.74841	28.8101	4.04246	6.76508	5.00E-05	0.0157425
Glyma.14G223400	(1 of 1) PTHR24413:SF79 - BTB/POZ DOMAIN-CONTAINING PROTEIN 9	control	*ppd-KO*	7.64453	28.7891	1.91302	4.19507	5.00E-05	0.0157425
Glyma.15G011000	(1 of 13) PF04862 - Protein of unknown function (DUF642) (DUF642)	control	*ppd-KO*	221.092	53.3239	-2.05179	-4.19706	0.00015	0.0420621
Glyma.15G017000	(1 of 6) PTHR11654:SF157 - PROTEIN NRT1/ PTR FAMILY 1.1-RELATED	control	*ppd-KO*	8.55381	29.6806	1.79488	3.93825	5.00E-05	0.0157425
Glyma.15G071300	(1 of 12) PTHR11772//PTHR11772:SF14 - ASPARAGINE SYNTHETASE	control	*ppd-KO*	1.32227	10.1815	2.94487	4.38812	0.00015	0.0420621
Glyma.15G072400	(1 of 12) PTHR11772//PTHR11772:SF14 - ASPARAGINE SYNTHETASE	control	*ppd-KO*	20.9513	127.772	2.60846	5.56653	5.00E-05	0.0157425
Glyma.15G108700	(1 of 3) 1.8.4.10 - Adenylyl-sulfate reductase (thioredoxin) / Thioredoxin-dependent 5'-adenylylsulfate reductase	control	*ppd-KO*	34.1685	10.8161	-1.65948	-3.63508	0.00015	0.0420621
Glyma.15G115000		control	*ppd-KO*	250.129	53.0913	-2.23613	-4.61098	5.00E-05	0.0157425
Glyma.15G166800	(1 of 309) PF00010 - Helix-loop-helix DNA-binding domain (HLH)	control	*ppd-KO*	2.19788	20.5537	3.22521	5.3967	5.00E-05	0.0157425
Glyma.16G089700	(1 of 172) KOG0156 - Cytochrome P450 CYP2 subfamily	control	*ppd-KO*	1.2416	13.0978	3.39905	5.42426	5.00E-05	0.0157425
Glyma.16G131200	(1 of 172) KOG0156 - Cytochrome P450 CYP2 subfamily	control	*ppd-KO*	1.69594	11.7915	2.7976	4.8322	5.00E-05	0.0157425

Glyma.16G188900	(1 of 11) 2.4.2.26 - Protein xylosyltransferase / Uridine diphosphoxylose-protein xylosyltransferase	control	*ppd-KO*	2.09335	0	-inf	NaN	5.00E-05	0.0157425
Glyma.16G190000	(1 of 29) PF00560//PF08263 - Leucine Rich Repeat (LRR_1) // Leucine rich repeat N-terminal domain (LRRNT_2)	control	*ppd-KO*	5.55979	0	-inf	NaN	5.00E-05	0.0157425
Glyma.16G193900	(1 of 117) PF00560//PF08263//PF13855 - Leucine Rich Repeat (LRR_1) // Leucine rich repeat N-terminal domain (LRRNT_2) // Leucine rich repeat (LRR_8)	control	*ppd-KO*	0.480463	0	-inf	NaN	5.00E-05	0.0157425
Glyma.16G212500	(1 of 50) PF00197 - Trypsin and protease inhibitor (Kunitz_legume)	control	*ppd-KO*	10.1521	107.265	3.40133	6.86182	5.00E-05	0.0157425
Glyma.16G214500	(1 of 2) PF13499//PF13676 - EF-hand domain pair (EF-hand_7) // TIR domain (TIR_2)	control	*ppd-KO*	1.69326	0	-inf	NaN	5.00E-05	0.0157425
Glyma.17G092800	(1 of 7) PTHR23201//PTHR23201:SF18 - EXTENSIN, PROLINE-RICH PROTEIN	control	*ppd-KO*	4.64785	45.5768	3.29367	6.07254	5.00E-05	0.0157425
Glyma.17G180400	(1 of 5) PTHR11709//PTHR11709:SF26 - MULTI-COPPER OXIDASE	control	*ppd-KO*	39.9198	8.7146	-2.1956	-4.77284	5.00E-05	0.0157425
Glyma.17G187600		control	*ppd-KO*	43.5048	187.386	2.10677	4.45185	5.00E-05	0.0157425
Glyma.17G203400	(1 of 20) PTHR10484 - HISTONE H4	control	*ppd-KO*	49.1141	0	-inf	NaN	5.00E-05	0.0157425
Glyma.18G116900	(1 of 3) 2.6.1.45 - Serine--glyoxylate transaminase / SGAT	control	*ppd-KO*	32.2393	6.22169	-2.37344	-4.96183	5.00E-05	0.0157425
Glyma.18G137800	(1 of 2) PTHR30544//PTHR30544:SF3 - 23S RRNA METHYLTRANSFERASE	control	*ppd-KO*	3.9659	0	-inf	NaN	5.00E-05	0.0157425
Glyma.18G141100	(1 of 6) 2.5.1.117 - Homogentisate solanesyltransferase / HST	control	*ppd-KO*	2.0824	0	-inf	NaN	5.00E-05	0.0157425
Glyma.18G142700	(1 of 1) PF00118//PF04078 - TCP-1/cpn60 chaperonin family (Cpn60_TCP1) // Cell differentiation family, Rcd1-like (Rcd1)	control	*ppd-KO*	0	2.87694	inf	NaN	5.00E-05	0.0157425

Glyma.18G143400	(1 of 5) PTHR19879:SF1 - CANNONBALL-RELATED	control	*ppd-KO*	0	3.55424	inf	NaN	5.00E-05	0.0157425
Glyma.18G182800	(1 of 1) PTHR12725//PTHR12725:SF68 - HALOACID DEHALOGENASE-LIKE HYDROLASE	control	*ppd-KO*	36.5535	127.275	1.79987	3.86395	0.00015	0.0420621
Glyma.18G222900	(1 of 17) 1.14.13.152 - Geraniol 8-hydroxylase / G10H	control	*ppd-KO*	1.83249	0	-inf	NaN	5.00E-05	0.0157425
Glyma.18G223100	(1 of 17) 1.14.13.152 - Geraniol 8-hydroxylase / G10H	control	*ppd-KO*	0.475865	0	-inf	NaN	0.0001	0.0296636
Glyma.18G225300	(1 of 17) PTHR32246:SF20 - BON1-ASSOCIATED PROTEIN 1-RELATED	control	*ppd-KO*	0	4.30065	inf	NaN	5.00E-05	0.0157425
Glyma.18G235700	(1 of 33) 2.4.1.34 - 1,3-beta-glucan synthase / UDP-glucose-1,3-beta-D-glucan glucosyltransferase	control	*ppd-KO*	6.79358	0	-inf	NaN	5.00E-05	0.0157425
Glyma.18G258700	(1 of 4) PTHR12565:SF172 - BHLH TRANSCRIPTION FACTOR PRE1-RELATED	control	*ppd-KO*	84.3793	19.1344	-2.14072	-4.52758	5.00E-05	0.0157425
Glyma.19G021400	(1 of 4) PTHR12565:SF172 - BHLH TRANSCRIPTION FACTOR PRE1-RELATED	control	*ppd-KO*	37.1492	3.60189	-3.3665	-5.57846	5.00E-05	0.0157425
Glyma.19G178200	(1 of 354) PF00847 - AP2 domain (AP2)	control	*ppd-KO*	3.55074	14.1778	1.99745	3.91577	5.00E-05	0.0157425
Glyma.20G040300	(1 of 21) PTHR23172 - AUXILIN/CYCLIN G-ASSOCIATED KINASE-RELATED	control	*ppd-KO*	0	1.18762	inf	NaN	5.00E-05	0.0157425
Glyma.20G144800	(1 of 2) PTHR12725//PTHR12725:SF81 - HALOACID DEHALOGENASE-LIKE HYDROLASE	control	*ppd-KO*	12.7749	84.3305	2.72274	5.66565	5.00E-05	0.0157425
Glyma.U004700	(1 of 6) PTHR11489 - 40S RIBOSOMAL PROTEIN SA	control	*ppd-KO*	0	0.808276	inf	NaN	5.00E-05	0.0157425
Glyma.U039500	(1 of 5) 4.2.1.70//5.4.99.12 - Pseudouridylate synthase / Uracil hydrolyase // tRNA pseudouridine(38-40) synthase / tRNA pseudouridylate synthase I	control	*ppd-KO*	0	2.80727	inf	NaN	5.00E-05	0.0157425

NaN, Not a Number; inf, positive infinite; -inf, negative infinite

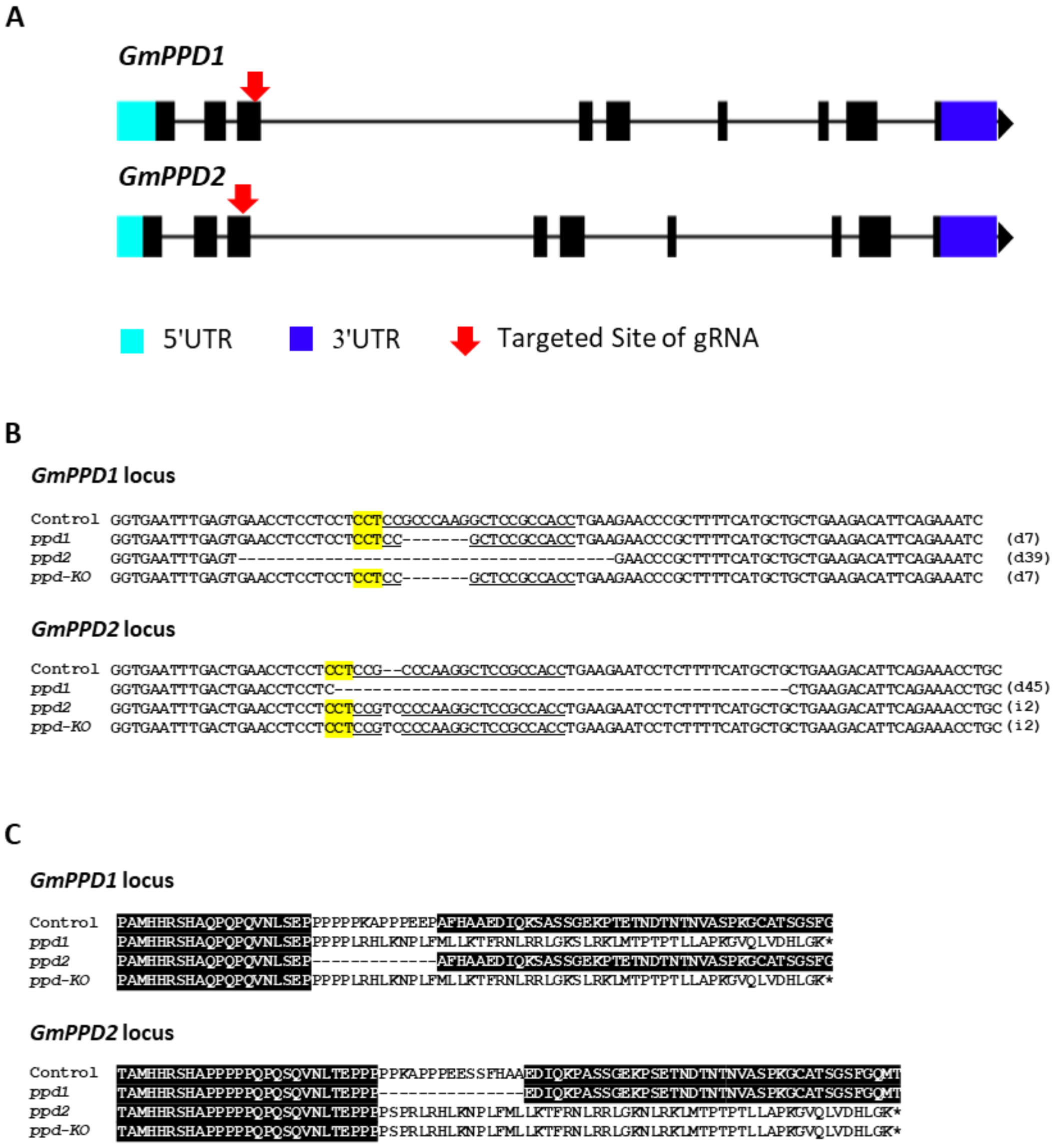

Supplementary Figure S1. Targeted region of gRNA, and nucleotide and predicted amino acid sequences of the targeted regions and its flanking region. (A) Schematic diagram of genome editing using the CRISPR/Cas9system in soybean. Boxes, exons; bold lines, introns. (B) Alignment of nucleotides at the targeted region in control plant and mutants. Nucleotide sequences of the targeted regions and its flanking region were determined by sequencing. Underlined sequences, targeted site of the gRNA; highlighted sequences, the proto-spacer regions; characters in parentheses, number of deleted or inserted nucleotides. (C) Alignment of predicted amino acid sequences at the targeted region in control plant and mutants. Shaded amino acid residues; same residues as control plant.

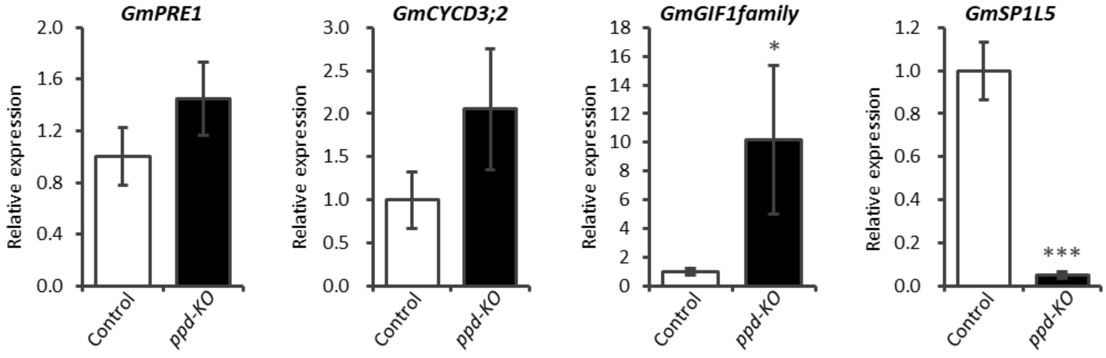

Supplementary Figure S2. Gene expression analysis of unifoliate leaves of the control and *ppd-KO* mutant plants. Relative gene expression level in unifoliate leaves of plants grown at 26°C for 14h light conditions. The expression of each gene was normalized to that of *Bic-C2* (Glyma.03G064800). * and *** indicate significant differences between control and the *ppd-KO* mutant plants at 5% and 0.1 % levels, respectively. All data shown are the mean ± SE of 3–4 biological replicates.
